# Integrative and deep learning-based prediction of therapy response in ovarian cancer

**DOI:** 10.1186/s13046-025-03554-w

**Published:** 2025-11-28

**Authors:** Alicja Rajtak, Ilona Skrabalak, Natalia Ćwilichowska-Puślecka, Agnieszka Kwiatkowska-Makuch, Marcin Poręba, Natalia Skrzypczak, Alicja Krasowska, Michael Pitter, Tomasz Maj, Jan Kotarski, Karolina Okla

**Affiliations:** 1 The First Department of Oncologic Gynecology and Gynecology, Medical University of Lublin, Lublin, Poland; 2 IOA, Lublin, Poland; 3 School of Medicine, Renji Hospital, Shanghai Jiao Tong University, Shanghai, China; 4 Department of Chemical Biology and Bioimaging, Faculty of Chemistry, Wroclaw University of Science and Technology, Wroclaw, Poland; 5 Department of Pathology, University of Michigan, Ann Arbor, MI USA; 6 Biomedical Engineering, University of Michigan Medical School, Ann Arbor, MI USA; 7 Department of Surgery, University of Michigan Medical School, Ann Arbor, MI USA; 8 Center of Excellence for Cancer Immunology and Immunotherapy, Rogel Cancer Center, University of Michigan, Ann Arbor, MI USA; 9 Pfizer, San Diego, CA USA

**Keywords:** Cancer, HGSOC, Tumor immunity, Tumor mutations, NK cells, TP53, TCF7, Therapy resistance, NGS, CyTOF, Machine learning

## Abstract

Ovarian cancer comprises a highly complex ecosystem of malignant cells and their surrounding tumor microenvironment (TME), where intricate interactions shape therapeutic responses. Most current predictive models fail to capture the full extent of these interactions. Here, we performed a comprehensive multi-omic analysis of pre-treatment ovarian tumor tissues, integrating clinical, genomic, transcriptomic, and immune features to correlate with pathological therapy response. Our results show that integrating genetic and immune parameters—particularly the interplay between NK cells and *TP53* status in high grade serous ovarian cancer (HGSOC), and diverse genetic alterations in non-HGSOC—markedly improves therapy response prediction. We demonstrate that tumor *TP53* status governs the persistence of early NK cells in HGSOC, and this persistent NK phenotype is associated with favorable clinical outcomes. Machine learning models harnessing these multi-omic features significantly outperform those based on any single information type alone. These findings highlight the central role of the baseline tumor ecosystem and support a precision oncology framework leveraging integrated multi-omic profiling and advanced analytics to improve prediction and guide treatment strategies.

**Supplementary Information:**

The online version contains supplementary material available at 10.1186/s13046-025-03554-w.

## Highlights

• Clinical features are insufficient predictors of therapy response.

• Genetic and immune features are linked to therapy response.

• Early NK cells exhibit a persistent phenotype associated with favorable clinical outcomes.

• Machine learning models integrating genomic and immune features significantly improve therapy response prediction.

• In HGSOC, therapy response depends on NK cell—TP53 interplay, while in non-HGSOC, it is driven by diverse genetic mutations.

• Tumor TP53 status regulates persistence of NK cells in HGSOC.

**Supplementary Information:**

The online version contains supplementary material available at 10.1186/s13046-025-03554-w.

## Introduction

Ovarian cancer is the most lethal gynecological malignancy and ranks as the eighth leading cause of cancer-related death among women. High-grade serous ovarian cancer (HGSOC) is the most prevalent histological subtype, accounting for 60–80% of all ovarian cancer cases [1]. Traditionally, treatment for advanced ovarian cancer has relied on cytoreductive surgery combined with platinum-based chemotherapy. Recent improvements in overall survival have been achieved with maintenance therapy using poly(ADP-ribose) polymerase (PARP) inhibitors—either as monotherapy or in combination with agents targeting VEGF, such as bevacizumab. Nonetheless, the outlook for patients with advanced-stage disease remains grim, with survival rates hovering around 25% at 5 years and 15% at 10 years post-diagnosis [2–5].

Notably, the use of immune checkpoint inhibitors (ICIs) has brought significant advancements in the treatment of many solid tumors, including most gynecological cancers except for ovarian cancer [2, 6]. However, the impact of ICIs on survival in ovarian cancer patients has been minimal, and as of now, no immunotherapy has gained regulatory approval for this type of cancer. Overall, despite advancements in surgery and chemotherapy, resistance to standard of care cytotoxic platinum-based therapies and immune evasion mechanisms remain major contributors to poor long-term survival in ovarian cancer [7–9]. While predictive biomarkers of therapy response have been identified through clinical, molecular, and digital pathology analyses, many previous studies have been constrained by single-platform profiling, which fails to fully capture the complexity of the tumor microenvironment (TME). As a result, chemotherapy selection continues to rely largely on empirical clinical risk stratification, based on factors such as tumor size, staging, and histological subtype.

Previously, we have shown that tumor ecosystem, comprising malignant clones and their dynamic interactions with immune components, is now recognized as a key determinant of the therapy response ecosystem [3, 8–16]. The growing understanding allows for predictive models to be improved by accounting for these complex TME. Notable, emerging evidence highlights that the mutational landscape is equally critical in ovarian cancer. Genetic alterations in key oncogenes and tumor suppressor genes not only drive tumor heterogeneity but also shape the immune microenvironment, influencing both immune evasion and therapeutic resistance [17–19]. For example, *TP53* mutations mediate tumor immune escape [20]*,* including attenuation of T cell and natural killer (NK) cell response and promotion of immunosuppressive myeloid-derived suppressor cells (MDSC), tumor-associated macrophages (TAMs) and regulatory T cells (Treg) [20–22].

Here, we performed a multi-omic analysis of a prospective treatment-naïve cohort, integrating clinical data, whole-exome sequencing (WES), RNA-sequencing (RNAseq) and tumor immune profiling using scRNAseq and mass cytometry (CyTOF). Our findings indicate that specific genetic alterations and immune infiltration patterns—particularly the interplay between NK cell populations and tumor *TP53* status in HGSOC, as well as diverse genetic aberrations in non-HGSOC—are strongly associated with therapy outcomes. Notably, we demonstrate that *TP53* mutations regulate the persistence of early NK cells in HGSOC, with this persistent NK phenotype linked to favorable clinical results. Leveraging these immunogenomic features, we developed machine learning models, showing that integrated models markedly outperform those based on any single data type. This integrative framework not only provides a robust approach for predicting therapy response across cancer types, but also supports the incorporation of novel biomarkers and data modalities—advancing precision oncology by more accurately reflecting the baseline tumor ecosystem and informing tailored treatment strategies.

## Results

### Overview of the study design

To investigate the clinical, genomic, and immune determinants of therapy response in ovarian cancer, we performed a comprehensive multi-omics analysis integrated with machine learning approaches. Pre-treatment tumor samples from patients with ovarian cancer and benign ovarian tumors were analyzed using next-generation sequencing (NGS) of DNA and RNA, mass cytometry (CyTOF), and clinical pathology. Patients were analyzed based on histological types including HGSOC and non-HGSOC. Patients were classified as responders or non-responders, with responders maintaining progression-free survival for at least six months following the completion of six cycles of platinum-based chemotherapy. Non-responders included patients with platinum-refractory disease, characterized by progression during the initial platinum-based chemotherapy, and those with platinum-resistant disease, defined by recurrence within six months of treatment completion (Supplementary Table 1). The identified features were incorporated into predictive models and validated in in silico modeling and functional studies.

**Fig. 1:**
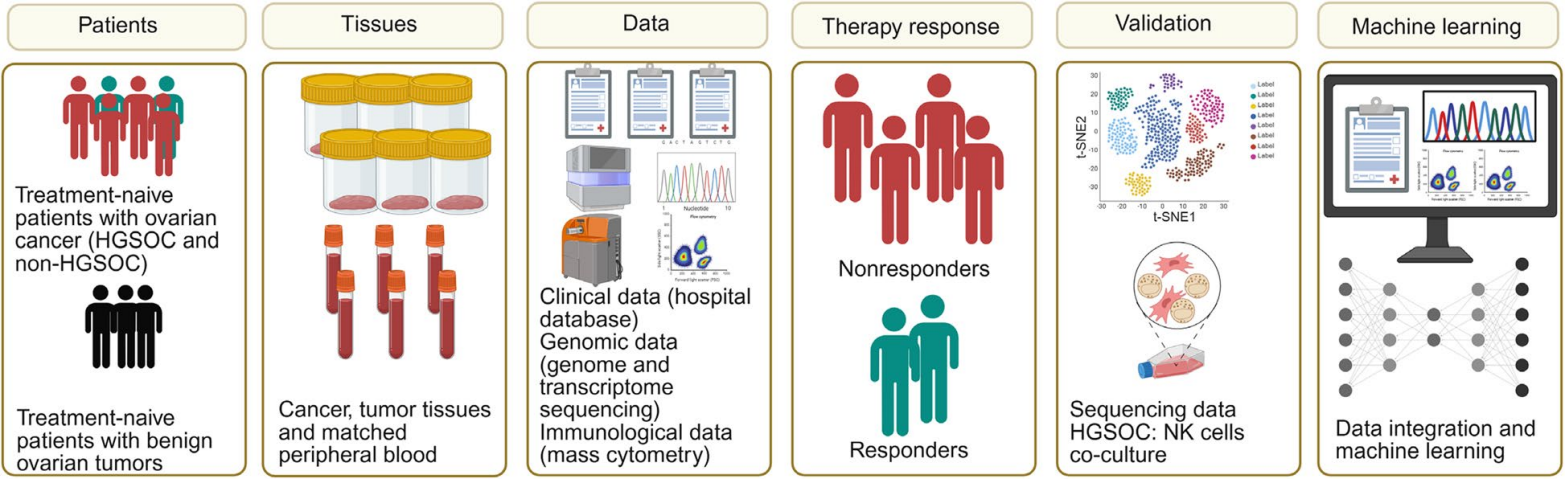
Overview of the study design. A cohort of treatment-naïve patients with ovarian cancer (*n* = 35) and benign ovarian tumors (*n* = 12) was selected for the study. Cancer and tumor tissue, along with peripheral blood samples, were collected from these patients. Clinical data were retrieved from hospital databases, while genomic data were obtained through Illumina genome sequencing. Immunological data were gathered using mass cytometry (CyTOF). The collected data were integrated and analyzed, with additional information from external databases, including RNA sequencing (RNAseq), single-cell RNA sequencing (scRNAseq) and high grade serous ovarian cancer (HGSOC) and natural killer (NK) cells co-culture system. Machine learning techniques were applied to identify patterns and correlations, leading to the classification of patients into responders and nonresponders. This approach was used to enhance the understanding of treatment responses and support the development of personalized therapeutic strategies for ovarian cancer.

Our study design (Fig. 1 and Figure S1) integrates multi-modal data, including patient-derived tumor samples, sequencing, immune profiling, clinical parameters, computational modeling, and functional studies to construct a comprehensive framework for understanding therapy response dynamics in ovarian cancer.

### Genomic alterations predict treatment outcomes beyond clinical features

We revealed that none of the evaluated clinical parameters, including tumor stage and tumor grade (Figure S2A, Supplementary Table 2), or commonly used serum biomarkers—cancer antigen 125 (CA125), human epididymis protein 4 (HE4) and the risk of ovarian malignancy algorithm (ROMA) score—were significantly associated with therapy response (Figure S2B-D) across all patients, as well as within HGSOC and non-HGSOC subgroups. In line with this, other factors including age, BMI and blood parameters were limited predictors (Supplementary Table 3).

Whole-exome sequencing (WES) of tumor samples (*n* = 35) identified a total of 146,243 somatic mutations Supplementary Table 4). Somatic mutation characteristics including copy number variations (CNVs), single nucleotide polymorphism (SNPs) and insertions/deletions (InDel) were comparable between responders and nonresponders (Figure S2E, Supplementary Table 5).

**Fig. 2:**
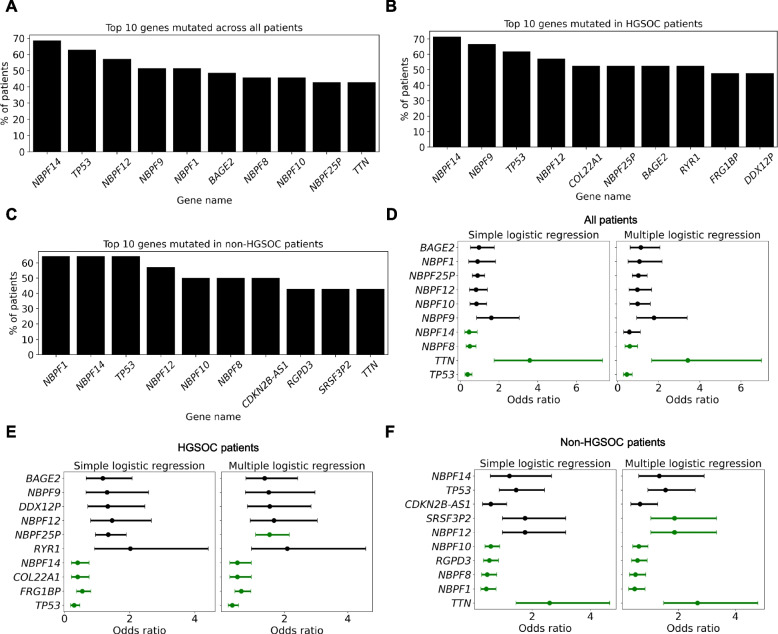
Genomic alterations predict treatment outcomes beyond clinical features. **A**-**C** Bar chart was used to show the top 10 most frequently mutated genes across all patients (**A**), high grade serous ovarian cancer (HGSOC) patients (**B**) and non-HGSOC patients (**C**). The x-axis was labeled with gene names, while the y-axis was used to indicate the percentage of patients with mutations in each gene (*n* = 35). **D**-**F** Simple and multiple logistic regression analysis was performed, and the odds ratios for various mutated genes were plotted for all patients (**D**), high grade serous ovarian cancer (HGSOC) patients (**E**) and non-HGSOC patients (**F**). The x-axis was used to represent the odds ratio, while the y-axis was labeled with the names of the analyzed genes. Some genes were highlighted in green, indicating that significant associations were found (*p* < 0.05; *n* = 35).

To further determine whether somatic genomic alterations can predict treatment response in ovarian cancer beyond established clinical features, we performed an integrative analysis of mutational profiles across all patients, as well as within HGSOC and non-HGSOC subgroups. We identified the ten most frequently mutated genes in each cohort (Fig. 2A-C) and assessed their association with treatment outcome using univariate (simple) and multivariate (multiple) logistic regression models (Fig. 2D-F).

Among all patients, the most frequently mutated genes included *NBPF14* (69%), *TP53* (63%), *NBPF12* (57%), and *NBPF9* (51%). Additional recurrently altered genes were *NBPF1*, *BAGE2*, *NBPF8*, *NBPF10*, *NBPF25P*, and *TTN* (Fig. 2A, Supplementary Table 6). The high prevalence of mutation within the NBPF gene family suggests this gene cluster may be broadly relevant to ovarian tumorigenesis or genome instability across subtypes. Using independent ovarian cancer cohort (TCGA-OV) we also confirmed high frequencies of mutational alterations in the NBPF gene family including *NBPF1*, *NBPF9*, *NBPF10*, *NBPF12* and *NBPF14* (> 50%) (Supplementary Table 7). In the HGSOC subset, the most frequently mutated genes were *NBPF14* (71%), *NBPF9* (67%), and *TP53* (62%). Other commonly altered genes included *NBPF12*, *COL22A1*, *NBPF25P*, *BAGE2*, and *RYR1*, *FRG1BP* and *DDX12P* (Fig. 2B, Supplementary Table 6). In contrast, the mutation landscape in non-HGSOC patients showed a distinct pattern. *NBPF1* (64%), *NBPF14* (64%), and *TP53* (64%) remained among the most mutated genes. Additional altered genes were *NBPF12*, *NBPF10*, *NBPF8*, *CDKN2B-AS1*, *RGPD3*, *SRSF3P2* and *TTN* (Fig. 2C, Supplementary Table 6). The divergent profiles between HGSOC and non-HGSOC patients prompted further investigation into the predictive value of these alterations for therapy response.

In univariate logistic regression including all patients, alterations in *TP53*, *NBPF8*, and *NBPF14* were significantly associated with decreased odds of therapy response (OR = 0.38, *p* = 0.00004; OR = 0.49, *p* = 0.0052; and OR = 0.45, *p* = 0.018, respectively), suggesting their potential role in mediating therapy resistance. Conversely, *TTN* alterations were strongly associated with increased odds of therapy response (OR = 3.59, *p* = 0.00049). In the multivariable model, which adjusted for co-occurring gene alterations, *TP53*, *NBPF8*, and *TTN* remained statistically significant (*TP53*: OR = 0.45, *p* = 0.00068; *NBPF8*: OR = 0.59, *p* = 0.036; *TTN*: OR = 3.41, *p* = 0.00081), indicating independent predictive effects (Fig. 2D, Supplementary Table 8).

In the HGSOC subgroup, *TP53* alterations were the most robust predictor of reduced therapy response (univariate OR = 0.30, *p* = 2.0e − 07; multivariate OR = 0.35, *p* = 6.0e − 06). Additionally, alterations in *FRG1BP*, *COL22A1*, and *NBPF14* were negatively associated with response (univariate ORs ranging from 0.41–0.55, all *p* < 0.005). These associations were retained in multivariate analysis (*FRG1BP*: OR = 0.64, *p* = 0.023; *NBPF14* and *COL22A1*: OR = 0.51, *p* = 0.035). Interestingly, *NBPF25P* emerged as a positive predictor in multivariate analysis (OR = 1.53, *p* = 0.015), suggesting a potential subtype-specific sensitization effect (Fig. 2E, Supplementary Table 8).

In contrast, *TP53* was not predictive of therapy response in the non-HGSOC subgroup (OR = 1.45, p = 0.16), indicating its predictive value may be restricted to HGSOC tumors. In this subgroup, multivariable analysis revealed that *TTN* was a strong and consistent positive predictor of response (OR = 2.67, *p* = 0.001). Negative predictors included *NBPF1*, *NBPF8*, *RGPD3*, and *NBPF10*, each associated with reduced odds of response (ORs 0.42–0.61, all *p* < 0.02). Moreover, *NBPF12* and *SRSF3P2* showed significant positive associations in multivariate analysis (OR = 1.86, *p* = 0.037), suggesting potential subtype-specific mechanisms (Fig. 2F, Supplementary Table 8).

These results highlight *TP53* as a subtype-specific negative predictor of therapy response, particularly in HGSOC, whereas *TTN* alterations were associated with increased response odds across non-HGSOC patients. Several *NBPF* family genes also emerged as potential modulators of therapy sensitivity, warranting further mechanistic exploration. Importantly, these findings underscore the heterogeneity of predictive biomarkers between ovarian cancer subtypes and may inform stratified therapeutic strategies.

### TME landscapes in ovarian tumors

**Fig. 3:**
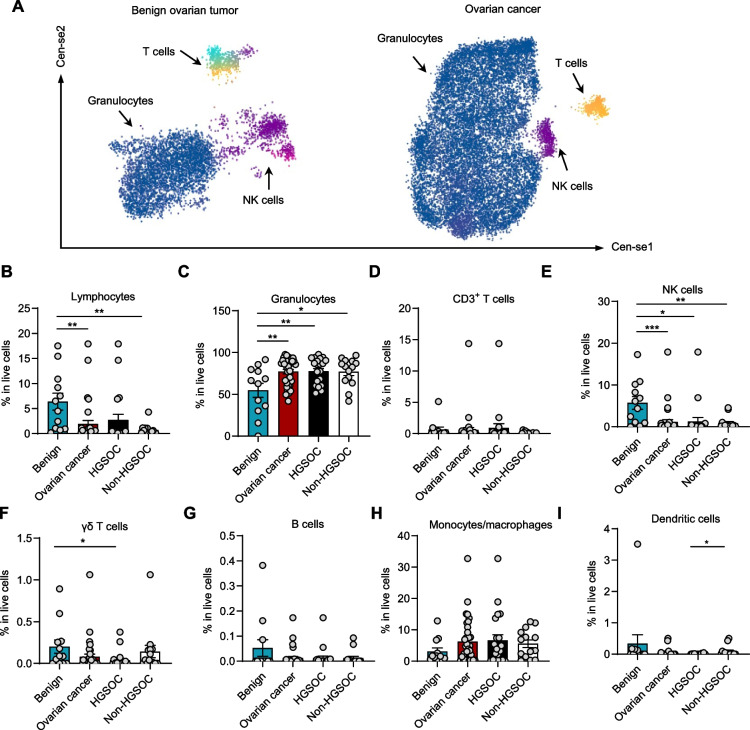
TME landscapes in ovarian tumors. **A** Two Cen-se plots of CyTOF display immune cell distributions in the TME of benign ovarian tumors (left) and ovarian cancer (right). Different immune cell populations, including granulocytes, T cells, and NK cells, were identified and color-coded. **B**-**I** The percentage of different immune cell populations, including lymphocytes (CD45⁺CD66b-) (**B**), granulocytes (CD45lowCD66b+) (**C**), CD3+ T cells (CD45+CD3+) (**D**), NK cells (CD45+CD56+) (**E**), gamma delta (γδ) T cells (CD4-CD8-TCRγδ+) (**F**), B cells (CD19+CD3-) (**G**), monocytes/macrophages (CD11c+HLA-DR+) (**H**), and dendritic cells (HLA-DR+) were analyzed using mass cytometry (CyTOF) (*n* = 47, mean ± SEM, ***p < 0.001, **p<0.01, *p < 0.05, unpaired t-test)

TME in therapy-naïve ovarian cancer and benign ovarian tumors remains poorly characterized, prompting a comprehensive analysis of its immune composition. To address this, we performed CyTOF profiling of tumor-infiltrating immune cell populations (Fig. 3A–I, Figure S3A and B), including lymphocytes (CD45⁺CD66b^−^) (Fig. 3B), granulocytes (CD45^low^CD66b^+^) (Fig. 3C), CD3^+^ T cells (CD45^+^CD3^+^) (Fig. 3D), NK cells (CD45^+^CD56^+^) (Fig. 3E), gamma delta (γδ) T cells (CD4^−^CD8^−^TCRγδ^+^) (Fig. 3F), B cells (CD19^+^CD3^−^) (Fig. 3G), monocytes/macrophages (CD11c^+^HLA-DR^+^) (Fig. 3H), and dendritic cells (HLA-DR^+^) (Fig. 3I). Gating strategy was shown in Figure S3. Cen-se analysis (Fig. 3A) and quantification of immune cell populations (Fig. 3B–I) revealed distinct immune landscapes between ovarian cancer and benign ovarian tumors.

We observed a reduction trend in total lymphocyte infiltration in ovarian tumor tissues—including both HGSOC and non-HGSOC subtypes—in comparison to benign ovarian lesions (*p* < 0.07; Fig. 3B). In parallel, the proportion of granulocytes among live cells was significantly increased in both HGSOC and non-HGSOC ovarian cancers relative to benign tissue (*p* < 0.001 and *p* < 0.01; respectively Fig. 3C). Assessment of T cell populations showed that the fraction of CD3⁺ T cells, while variable among individual samples, remained broadly comparable between benign and malignant tissues (Fig. 3D). In contrast, NK cell infiltration was markedly depleted in ovarian tumors in comparison to benign samples, a pattern seen consistently in both major ovarian cancer subtypes (Fig. 3E; *p* < 0.01 and *p* < 0.001). Further, the proportion of γδ T cells was modestly but significantly depleted in HGSOC tumors (*p* < 0.05; Fig. 3 F), while frequencies of B cells (Fig. 3G) and monocytes/macrophages (Fig. 3H) remained comparable between groups. Notably, dendritic cell infiltration was slightly decreased in HGSOC compared to non-HGSOC (*p* < 0.05; Fig. 3I) (Supplementary Table 9).

Taken together, these results reveal that malignant transformation in ovarian tissue is accompanied by a selective depletion of total lymphocytes and NK cells, combined with a relative enrichment of granulocytes. This profound remodeling of the TME may contribute to immune evasion and tumor progression in ovarian cancer. Specifically, insufficient infiltration of anti-tumor NK cells may contribute to immune evasion in ovarian cancer, which was previously reported [8, 23, 24], underscoring the importance of TME composition in shaping immune surveillance in ovarian cancer.

### Tumor early NK cells dictate clinical outcome in ovarian cancer

**Fig. 4:**
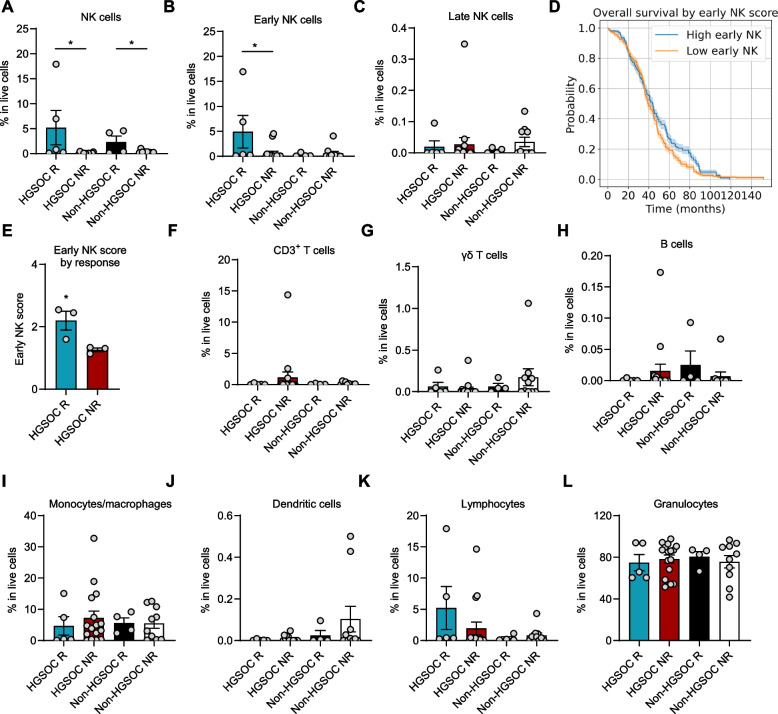
Tumor early NK cells dictate clinical outcome in ovarian cancer. **A**-**C** Mass cytometry (CyTOF) analysis of NK cells (**A**) early NK cells (**B**) and late NK cells (**C**) show in responders (R) and nonresponders (NR) across high grade serous ovarian cancer (HGSOC) patients and non-HGSOC patients (*n* = 35, mean ± SEM, *p < 0.05, unpaired t-test). **D** Kaplan-Meier survival curve evaluates the impact of high and low early NK cell score in HGSOC patients using data from the TCGA-OV dataset (*n* = 429, log-rank *p* = 0.00003). Early NK cell score was assessed using deconvolution method. **E** The level of NK score by therapy response in HGSOC patients (*n* = 6, mean ± SEM, *p < 0.05, unpaired t-test). HGSOC tissues were sequenced (RNAseq) and early NK score was assessed using deconvolution method. **F**-**L** CyTOF analysis of CD3+ T cells (**F**), gamma delta (γδ) T cells (**G**) B cells (**H**), monocytes/macrophages (**I**), and dendritic cells (**J**), lymphocytes (**K**) and granulocytes (**L**) show in responders (R) and nonresponders (NR) across HGSOC patients and non-HGSOC patients (*n* = 35, mean ± SEM)

We hypothesized that TME plays a critical role in predicting therapy response. Although certain components of the TME have been shown to influence chemotherapy response in ovarian cancer [25], yet the detailed characteristics distinguishing tumors in responders from those in nonresponders remain largely undefined. To further investigate how immune dysfunction contributes to therapy resistance, we stratified patients into responders and non-responders and analyzed tumor infiltration by immune cell subsets in the HGSOC and non-HGSOC groups (Fig. 4A-C and F-L).

We first observed an enrichment of early NK cells (CD56^+^CD57^−^) (*p *< 0.001) (Figure S4A) in benign ovarian tumors compared to ovarian cancer, while late NK cell levels did not differ significantly (Figure S4B). This prompted a focused analysis of NK cell populations in therapy-responsive and non-responsive ovarian cancer tumors. Quantification of tumor NK cells demonstrated a significantly lower proportion of NK cells in non-responder tumors compared to responders in both HGSOC and non-HGSOC subtypes (*p* < 0.05; Fig. 4A and Figure S4C). This reduction was primarily attributable to a marked depletion of early NK cells in non-responders (*p* < 0.05; Fig. 4B) in HGSOC group but not HGSOC (Fig. 4B). In contrast, proportion of late NK cells were comparable between groups (Fig. 4C) (Supplementary Table 10). Next, using RNAseq data from independent HGSOC cohort (TCGA-OV) we performed cell-type deconvolution [26] for early NK cells and stem-like CD8^+^ T cells. Survival analysis revealed a robust association between early NK cell infiltration and clinical outcome; patients stratified as having high tumor early NK cell scores exhibited significantly improved overall survival compared to those with low tumor early NK cell scores (log-rank *p* = 0.033; Fig. 4D). Notably, stem-like CD8^+^ T cell score was not associated with overall survival improvement (Figure S4D). To validate these results, we performed RNAseq and deconvolution for early NK cells using our patient cohort. Consistently, the median early NK cell score was significantly higher in HGSOC responder tumors than in non-responders (*p* < 0.05; Fig. 4E).

The proportions of other major immune cell subsets—including CD3⁺ T cells (Fig. 4F), γδ T cells (Fig. 4G), B cells (Fig. 4H), monocytes/macrophages (Fig. 4I), dendritic cells (Fig. 4J), lymphocytes (Fig. 4K), and granulocytes (Fig. 4L) were comparable between responder and non-responder tumors, highlighting the unique clinical relevance of early NK cell infiltration in HGSOC patients.

Together, these findings demonstrate that early NK cell infiltration is uniquely associated with therapy response and favorable survival specifically in HGSOC tumors.

### Tumor early NK cells show persistent functional phenotype

To further characterize the NK cell immune landscape in ovarian cancer, we analyzed tumor-infiltrating immune cells using single-cell RNA sequencing (scRNA-seq) data from the GSE180661 dataset [18] including 42 treatment-naive patients. UMAP (uniform manifold approximation and projection) clustering identified 14 distinct cellular clusters using the Seurat pipeline (Figure S5A). Marker gene expression analysis confirmed the presence of CD8⁺ T cells (*CD8A*), CD4⁺ T cells (*CD4*), and NK cells (*NKG7*, *NCAM1*, *KLRD1*, and *KLRF1*) (Figure S5B). Among these clusters, NK cells were distinctly represented in clusters 2, 8, 12, and 14 (Figure S5A and B).

**Fig. 5:**
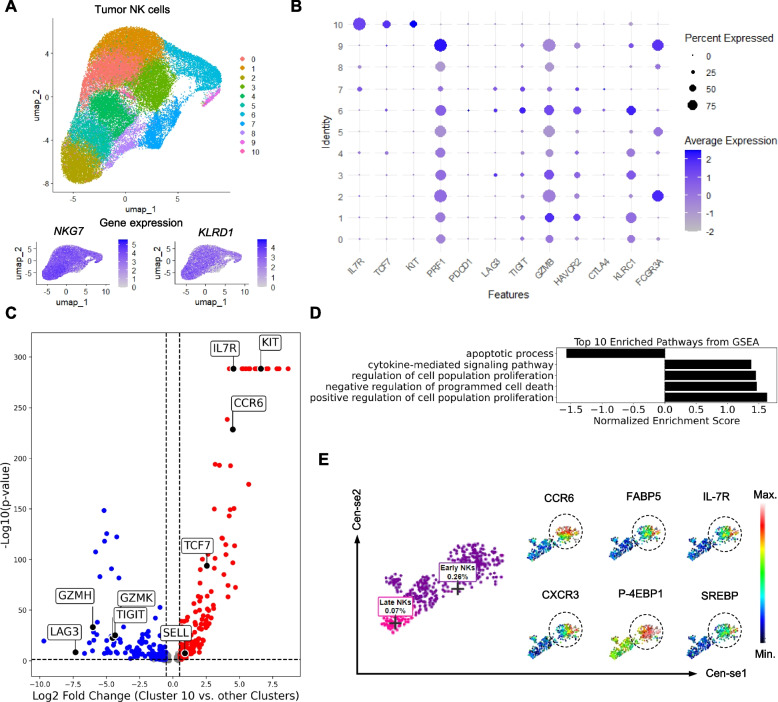
Tumor early NK cells show persistent phenotype. **A** Uniform manifold approximation and projection (UMAP) clustering of NK cells were shown, where NK cells are grouped into different clusters, each represented by a distinct color. Below the UMAP plot, two smaller density plots display the expression of *NKG7 *and *KLRD1*, which are key markers associated with NK cells (*n* = 42 HGSOC patients, GSE180661). **B** A dot plot displaying gene expression across different NK cell clusters. The dot size represents the percentage of cells expressing a particular gene, while the color intensity indicates average expression level. **C** A volcano plot illustrating differentially expressed genes between early-like NK cells (cluster 10) and other clusters (late-like NK cells). Red points represent upregulated genes (e.g., *IL7R, KIT, CCR6*), while blue points indicate downregulated genes (e.g., *GZMK, LAG3, TIGIT*) (*p* < 0.05). **D** A bar graph showing the enriched biological pathways from gene set enrichment analysis (GSEA) between early-like NK cells (cluster 10) and other clusters (late-like NK cells). **E** Mass cytometry (CyTOF) Cen-se plot illustrates the separation of early NK cells and late NK cells, with the early NK cell population distinctly marked. Six additional small plots display the expression patterns of different proteins, including CCR6, CXCR3, FABP5, P-4EBP1, IL-7R, and SREBP. A heatmap-like color gradient was used, where high expression is represented in red and low expression in blue, indicating the differential expression of these markers in various NK cell populations

Next, to focus specifically on NK cell heterogeneity, we excluded non-NK cell clusters and reanalyzed only tumor-infiltrating NK cells. UMAP clustering revealed 11 distinct NK cell subpopulations (Fig. 5A). A dot plot visualization of immune regulatory gene expression demonstrated that *IL7R*, *KIT*, and *TCF7*, genes associated with NK cell activation and survival, were differentially expressed across clusters (Fig. 5B). The observed variability in gene expression suggests functional heterogeneity within the NK cell compartment, with certain subsets displaying a more persistent activation phenotype.

To further investigate NK cell persistence, we performed differential gene expression analysis comparing early NK cells (Cluster 10) with other NK cell clusters. A volcano plot revealed significant upregulation of *IL7R*, *KIT*, *CCR6*, *TCF7*, and *SELL* in early NK cells, suggesting their role in early activation and prolonged immune function. In contrast, genes associated with cytotoxicity and immune exhaustion, including *GZMK*, *LAG3*, and *TIGIT*, were significantly downregulated in this population (Fig. 5C). These findings indicate that early NK cells exhibit a functionally distinct phenotype characterized by sustained activation and reduced exhaustion.

Gene set enrichment analysis (GSEA) identified key biological pathways enriched in early NK cells, including cytokine-mediated signaling, cell population proliferation, and negative regulation of cell death (Fig. 5D). These pathways suggest that early NK cells play a critical role in maintaining immune surveillance and may contribute to effective anti-tumor responses.

To evaluate the functional properties of NK cells, we analyzed key markers using CyTOF (Fig. 5E). Antibodies against FABP5, P-4EBP1, and SREBP were conjugated to distinct metal isotopes (116Cd, 162Dy, and 165Ho, respectively) to enable simultaneous detection without spectral overlap. CyTOF analysis revealed that early-activated NK cells exhibited enhanced expression of CCR6, FABP5, IL7RA, CXCR3, P-4EBP1, and SREBP. In contrast, late NK cells displayed features consistent with terminal differentiation, suggesting an impaired immune response (Fig. 5E).

Collectively, these findings indicate that NK cell persistence may serve as a potential biomarker for stratifying patients based on their likelihood of therapy response. The distinct molecular and functional properties of early NK cells highlight their role in immune regulation and their potential as a therapeutic target in ovarian cancer.

### Integrating composite machine learning techniques to enhance therapy response predictions

**Fig. 6:**
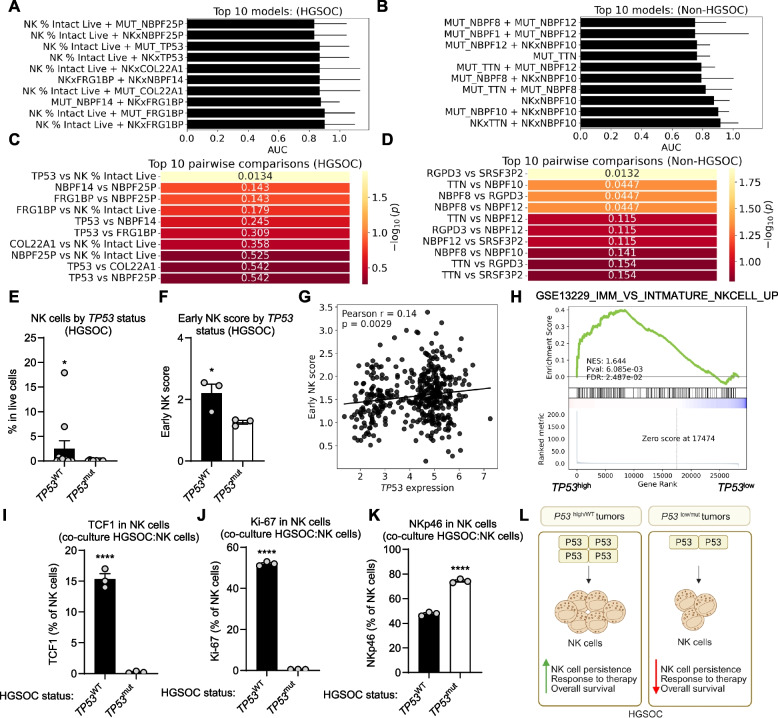
Integrating composite machine learning techniques to enhance therapy response predictions. **A** and **B** Top 10 composite machine learning models in predicting therapy response in high-grade serous ovarian cancer (HGSOC) (**A**) and non-HGSOC (**B**) ranked by are under curve (AUC) (*n* = 35). **C** and **D** Top 10 pairwise feature comparisons contributing to therapy response prediction in HGSOC (**C**) and non-HGSOC (**D**) (*n* = 35). **E** Mass cytometry (CyTOF) analysis of percentage of NK cells by *TP53 *status in HGSOC patients (*n* = 21). **F** The level of early NK score by *TP53 *status (*TP53*WT; wild type *TP53 *and *TP53*mut; mutated *TP53*) in HGSOC patients (*n* = 6, mean ± SEM, *p < 0.05, unpaired t-test). HGSOC tissues were sequenced (RNAseq) and early NK score was assessed using deconvolution method. **G** Correlation of *TP53 *expression with early NK cell score across HGSOC patients (*n* = 382, TCGA-OV). Early NK score was assessed using deconvolution method. **H** Gene set enrichment analysis (GSEA) plot showing enrichment of the IMM_VS_INTMATURE_NKCELL signature between *TP53*-high and *TP53*-low expression groups (*n* = 6, HGSOC). **I**-**K** Flow cytometry analysis of TCF7 (**I**), Ki-67 (**J**), NKp46 (**K**) in natural killer (NK) cells. Human NK cells (YT) were co-cultured with high grade serous ovarian cancer (HGSOC) cells (*TP53*WT; wild type *TP53 *(CAOV3) and *TP53*mut; mutated *TP53 *(COV362)) (*n* = 6, mean ± SEM, ****p < 0.00001, unpaired t-test, one from three experiments is shown). **L** Working model. *TP53 *increases infiltration and functional activity of NK cells, resulting in better responses to therapy and improved overall survival. In contrast, *TP53*mut tumors are characterized by reduced persistence of NK cells leading to impaired immune surveillance, worse therapy outcomes, and shorter survival

To comprehensively examine the determinants of therapy response, we implemented integrative machine learning models in both HGSOC and non-HGSOC tumor cohorts. Across both datasets, composite models that incorporated immune component (NK cells) in tandem with mutational profiles (including *TP53*, *TTN*, *FRG1BP*, *COL22A1*, *RPGD3*, *NBPF14*, *SRSF3P2*, *NBPF25P*, *NBPF12*, *NBPF10*, *NBPF8*, and *NBPF1* status) robustly outperformed single-parameter classifiers, as reflected by higher AUC values among the top models (Fig. 6A and B, Supplementary Table 11). Notably, while in the HGSOC cohort top predictive pairs involved NK cells and *TP53* mutation status (Fig. 6C, Supplementary Table 12), in non-HGSOC tumors distinctive combinations, such as signatures involving *RGP3D* and *SRSF3P2* genes, were dominant (Fig. 6D, Supplementary Table 12), thus underscoring tissue-specific interplay in immune surveillance and genetic context.

Inspired by these results, we investigated whether *TP53* affects NK cells in HGSOC. We divided HGSOC patients based on *TP53* mutational status (*TP53*^WT^; wild type *TP53* and *TP53*^mut^; mutated *TP53*) and observed that tumors harboring *TP53* mutations showed a substantial reduction of NK cells (Fig. 6E). To further validate these results, we performed RNAseq and next deconvolution for early NK cells. Similarly, we showed that early NK score was diminished in the *TP53*^mut^ group (Fig. 6F). In line with this, using our HGSOC patient cohort (Figure S6A) and external HGSOC patient cohort (TCGA-OV database) (Fig. 6G), we demonstrated a strong positive correlation between *TP53* transcript levels and early NK score. Concordantly, Kaplan–Meier estimates highlighted inferior overall survival in the subgroup of patients with low *TP53* expression (Figure S6B). These findings were mirrored at the transcriptional level, where GSEA consistently revealed that samples with high *TP53* expression are enriched for genes characteristic of immature NK cell phenotypes (Fig. 6H and Figure S6C).

To validate these bioinformatic predictions, we conducted co-culture experiments, which confirmed that NK cells exposed to *TP53*^mut^ HGSOC environments had significantly reduced expression of stem-like transcriptional factor TCF1 [27] (Fig. 6I), proliferation (Fig. 6J), as well as increased oxidative stress, as measured by MitoSOX levels (Figure S6D). Moreover, level of NK cell receptor which can be related to terminal differentiation NKp46 [28, 29] was upregulated in *TP53*^mut^ group (Fig. 6K).

Together, our results reveal that in HGSOC, the *TP53*-driven loss of NK cell fitness mediates diminished anti-tumor immunity and therapy response, contributing to an adverse clinical outcome (Fig. 6L).

## Discussion

### Ovarian cancer as a complex tumor ecosystem

Ovarian tumors represent highly complex ecosystems, comprising heterogeneous malignant cell populations and a dynamic TME that includes immune components [3, 8, 9, 11, 24]. The functional organization of these ecosystems is closely linked to their genomic and immune landscapes, with therapy-induced perturbations playing a pivotal role in determining treatment response [30]. However, previous efforts to identify pre-treatment predictors of therapy response have often overlooked these intricate interactions.

### Genomic and immune determinants of therapy response

The most important findings of this study can be highlighted. First, our findings indicate that pre-treatment genomic alterations and TME features offer superior prediction of therapy response in ovarian cancer compared to traditional clinical parameters and serum biomarkers. Neither tumor stage, grade, nor commonly used markers (CA125, HE4, ROMA) were associated with treatment outcomes across our cohort. Second, our results show that genomic predictors of therapy response differ between ovarian cancer subtypes. In HGSOC, *TP53* mutations strongly predicted poor therapy response, while in non-HGSOC, *TTN* mutations were positively associated with treatment efficacy; *TP53* was not predictive in this group. *NBPF* gene alterations also showed subtype-specific associations, highlighting the molecular diversity of ovarian cancers. Third, early NK cell infiltration in the TME was consistently linked to improved therapy response and survival in HGSOC. These findings support integrating molecular and immune profiling—tailored to tumor subtype—into personalized ovarian cancer therapy. The identification of early NK cells as a key predictor of response underscores their potential as both a biomarker and a therapeutic target.

### Machine learning-based prediction of therapy response

Further, our findings show that integrative machine learning models combining genomic and immune features outperform single-parameter models in predicting therapy response in ovarian cancer, with subtype-specific determinants of predictive power. In HGSOC, the strongest predictors were combinations of NK cell abundance and *TP53* mutation status, highlighting the interplay between *TP53*-driven immune dysfunction and therapeutic resistance. In contrast, for non-HGSOC tumors, the best-performing models relied on unique combinations of different gene mutations (such as *RGP3D* and *SRSF3P2*) rather than immune components. Mechanistically, we found that *TP53* mutations in HGSOC impair NK cell function and abundance, providing a link between genetic alterations, immune evasion, and poor therapy response. These results underscore the need for tailored, multi-omic predictive models adapted to ovarian cancer subtype to better guide personalized treatment strategies.

### Study limitations

Despite its key insights, this study has several limitations. The moderate sample size may not fully capture the extent of tumor heterogeneity, necessitating larger multi-center validation efforts. The reliance on single time-point sampling restricts the ability to assess tumor evolution over time, emphasizing the need for longitudinal studies. Moreover, cross-dataset comparisons may be influenced by residual batch effects despite standard normalization procedures. While our approach integrates genomic and immune features, additional modalities such as spatial transcriptomics could provide deeper mechanistic insights. Further, functional validation of key findings is essential to establish causality. Although the machine learning model demonstrated high predictive accuracy, its generalizability across different platforms and clinical settings requires further external validation in the prospectively collected patients’data.

Future research should focus on prospective validation in larger patient cohorts, functional studies to elucidate the role of key predictive markers, and real-world clinical applications to enhance precision oncology strategies in ovarian cancer.

### Clinical implications and future directions

Our study underscores the need to move beyond traditional clinical predictors by integrating genomic and immune profiling—especially early NK cells and key gene mutations—for more accurate therapy response prediction in ovarian cancer. Machine learning models that combine these features offer a robust framework for personalized treatment decisions. Additionally, targeting NK cell dysfunction, for example through engineering chimeric antigen receptor (CAR)-NK [31–34] cell therapies, may open new therapeutic avenues. Future work should focus on validating these findings in larger cohorts and advancing integrated predictive tools into clinical practice to improve patient outcomes.

## Methodology

### Patients

Treatment-naïve patients (*n* = 50) were prospectively enrolled at the 1 st Department of Oncological Gynecology and Gynecology and 2nd Chair and Department of Gynecology at Medical University of Lublin. Tumor tissues were collected during primary debulking surgeries. 12 patients with benign ovarian tumors (reference group) and 35 patients with ovarian cancer were analyzed (n = 21 HGSOC; *n* = 14 non-HGSOC). 3 patients were excluded due to the lack of complete biological material. The study was approved by the ethics committee of the Medical University of Lublin. Each participant provided written informed consent in accordance with the Declaration of Helsinki, and all experiments were conducted in compliance with ethical standards. The nature and possible consequences of the study were explained in detail to be forehead all participants.

Participants were recruited based on suspected diagnosis of ovarian cancer. The study included both patients with ovarian cancer and diagnosed with benign ovarian tumors. Inclusion criteria were age above 18 years and histologically confirmed diagnosis of ovarian cancer or benign ovarian tumor. Exclusion criteria for patients were serious intercurrent acute or chronic illnesses, presence of infections, allergic, or autoimmune disorders, presence of concurrent malignancies other than ovarian cancer and benign ovarian tumors, previous anticancer therapy prior to surgery and use of immunosuppressant drugs.

The pathological characteristics of the tumors were classified according to the World Health Organization (WHO) criteria, while the clinical stage was determined based on the International Federation of Gynecology and Obstetrics (FIGO) system. Tumor grading followed the Shimizu-Silverberg classification. Preexisting clinical data including protein biomarker levels of CA125, HE4 and ROMA in the blood were collected from a centralized database. The characteristics of patients are described in the Supplementary Table 1.

### Sample processing

Biological material including tumor tissue and peripheral blood was collected from patients and processed immediately after surgical excision. Peripheral blood samples were collected for mononuclear cell isolation. Whole blood was layered onto Gradisol (AquaMed) in a 2:1 ratio and centrifuged at 450 rcf for 5 min at room temperature (RT). The mononuclear cell layer (buffy coat), which formed at the interphase, was carefully collected for further analysis. Tumor tissue specimens were washed in PBS and divided into portions for downstream applications. For NGS – one portion of the tumor was chopped into small fragments (~ 1 mm^3^) and preserved in RNAlater (Thermo Fisher Scientific) for genome sequencing. For mononuclear cell isolation – the second portion of the tumor was mechanically homogenized and subjected to density gradient centrifugation on Gradisol (AquaMed), following centrifugation conditions (450 rcf for 20 min, RT). The mononuclear cell layer was collected. Cryopreservation was performed in fetal bovine serum (FBS) (PAN Biotech, Aidenbach, Germany) supplemented with 10% dimethyl sulfoxide (DMSO) (Sigma-Aldrich) and stored at − 80 °C until further use.

### DNA sequencing

A total of n = 70 samples (n = 35 peripheral blood, n = 35 tumor tissues) from patients with ovarian cancer were processed for DNA sequencing. Tissue samples were initially dissected into small fragments and immediately snap-frozen in liquid nitrogen to preserve DNA integrity. Genomic DNA was extracted using a commercial DNA extraction kit following the supplier’s standardized protocol. The extracted DNA was assessed for quality and concentration using Qubit Fluorometer (Invitrogen), while DNA integrity was analyzed using Agilent Bioanalyzer. Real-time PCR was performed to evaluate DNA concentration and amplification efficiency. DNA was then randomly fragmented to lengths of 180–280 bp, followed by end-repair, A-tailing, ligation of Illumina adapters, and PCR amplification. Fragment size selection was performed using AMPure XP magnetic beads (Beckman Coulter) to obtain the desired length range. For whole exome sequencing (WES), the libraries underwent hybridization-based enrichment with biotinylated probes, followed by washing, removal of unbound fragments, and enzymatic digestion of probes before a final PCR amplification step. The prepared libraries were pooled based on the required sequencing depth and DNA concentration and sequenced on an Illumina platform using paired-end 150 bp (PE150) reads. After sequencing, raw data were processed through several bioinformatics pipelines, including quality control (QC), read filtering, and adapter removal. The clean reads were then aligned to the reference genome using the Burrows-Wheeler Aligner (BWA), and single nucleotide polymorphism (SNP) and indel identification were performed using GATK. Variant filtering and annotation were applied, followed by the analysis of somatic variants in tumor samples. The sequencing statistics were evaluated, including read depth, genome coverage, and Q30 scores, where a Phred score > 85% was required for downstream analysis.

### RNA sequencing

A total of 6 HGSOC were processed for RNA sequencing. Tissue samples were dissected into small fragments and immediately snap-frozen in liquid nitrogen to preserve RNA integrity. Total RNA was extracted using a commercial RNA extraction kit, following the manufacturer’s standardized protocol. Messenger RNA was purified from total RNA using poly-T oligo-attached magnetic beads. After fragmentation, the first strand cDNA was synthesized using random hexamer primers, followed by the second strand cDNA synthesis using either dTTP for non-strand specific library or dUTP for strand specific library. The library was checked with Qubit and real-time PCR for quantification and bioanalyzer for size distribution detection. After library quality control, different libraries were pooled based on the effective concentration and targeted data amount, then subjected to Illumina sequencing. Rawdata (raw reads) of fastq format were firstly processed through fastp software. In this step, clean data (clean reads) were obtained by removing reads containing adapter, reads containing ploy-N and low-quality reads from raw data. At the same time, Q20, Q30 and GC content the clean data were calculated. All the downstream analyses were based on the clean data with high quality. Reference genome and gene model annotation files were downloaded from genome website. Use HISAT2 (2.2.1) to build the index of the reference genome and use HISAT2 to align paired-end clean reads to 2 the reference genome. FeatureCounts (2.0.6) was used to count the reads numbers mapped to each gene. And then FPKM of each gene was calculated based on the length of the gene and reads count mapped to this gene.

### Mass cytometry (CyTOF)

Maxpar Direct Immune Profiling Assay (Standard Biotools) was used for tumor immune cell profiling.

SREBP, P4EBP1, FABP5 antibodies were pre-conjugated with metals using the Maxpar® Antibody Labeling Kit (Fluidigm) according to the manufacturer’s instructions. Antibodies had a starting concentration of 0.5 mg/mL and intracellular antibody concentration was determined based on titration on PBMC. Iridium for incubation with cells was diluted in Fix and Perm Buffer (Standard Biotools). Initial concentration of iridium r-r (Standard Biotools) was 125 µM and used 1 µL + 1 mL Fix and Perm Buffer was used. The cells were centrifuged at 1800 rcf for 5 min at RT, and the pellet was blocked in 1 mm of 5% bovine serum albumin (BSA) for 30 min at RT to reduce non-specific antibody binding. Cells were then washed twice with PBS and Cell Staining Buffer (CSB) and centrifuged under the same conditions (1800 rcf, 5 min, RT). The washed cells were subsequently incubated with the Maxpar Direct Immune Profiling Assay panel for 30 min to allow surface marker staining. Following this incubation, the cells were washed again with CSB and centrifuged 1800 rcf, 5 min, RT. Next, the cells underwent fixation and permeabilization using the Foxp3 Fixation/Permeabilization Assay. For this purpose, 1 mL each of Fixation/Permeabilization Concentrate and Fixation/Permeabilization Diluent were mixed, and 0.5 mL of working solution (WS) was added for 30 min in the dark. After incubation, 0.5 mL of Permeabilization Buffer was added, and the cells were centrifuged at 1800 rcf for 8 min at RT. The pellet was then blocked again in 1 mL of 5% BSA for 30 min at RT, followed by another washing step with CSB and centrifugation (1800 rcf, 5 min, RT). The cells were then subjected to intracellular staining with a cocktail of metal-conjugated antibodies suspended in CSB. The following antibodies were used: ACADM, VDAC1, CPT1a, GLUT1, P4EBP1, FABP5, and SREBP, each added at a concentration of 1 µL per 50 µL of CSB. Staining was performed for 30 min at RT. After staining, the cells were washed with CSB, centrifuged (1800 rcf, 5 min, RT), and 1 mL of 1.6% formaldehyde (FA) was added for 10 min in the dark to fix the cells. The cells were centrifuged (1800 rcf, 6 min, RT) and then resuspended in 1 mL of Iridium (Intercalator-Ir) solution for 12 h at 4 °C to allow DNA labeling. This step ensures the identification of nucleated cells and exclusion of debris or doublets during mass cytometry acquisition. Cells were then resuspended in an appropriate volume of Beads Solution to achieve a final concentration of 500,000 cells/mL at a 1:9 ratio. The samples were filtered through a 40 µM filter to remove cell aggregates and ensure optimal sample integrity. Finally, the prepared samples were acquired using CyTOF-Helios (Fluidigm).

Gating strategy was presented at the Fig. S3. Initial pre-processing involved exclusion of calibration beads and removal of acquisition artifacts by gating on parameters such as residual, center, offset, width, and event length over time. Next, cell viability was assessed by selecting Live/Dead-negative events to exclude dead cells. Singlet discrimination was performed by gating on DNA1 and DNA2 signals, allowing the selection of intact, nucleated single cells and removal of cell debris and doublets.

CD45 expression was used to identify total leukocytes, and CD66b expression helped distinguish granulocytes (CD66b^+^) from lymphocytes and mononuclear cells (CD45^+^CD66b^−^). From this point, specific immune cell subsets were gated within the CD45^+^CD66b^−^ population. B cells were identified as CD19^+^CD3^−^ cells. γδ T cells were identified based on CD3 and TCRγδ co-expression (CD3^+^TCRγδ^+^). NK cells were gated as CD45^+^CD56^+^ and further divided into early NK cells (CD56^+^CD57^−^) and late NK cells (CD56^+^CD57^+^). Monocytes and macrophages were gated as CD11c^+^HLA-DR^+^ Dendritic cells were defined as HLA-DR^+^.

### Flow cytometry

Surface marker staining was performed by incubating human NK cells (YT cells) with the appropriate antibodies for 30 min (anti-CD56). For intracellular staining, cells were washed, resuspended in Fix/Perm buffer (BD Biosciences), and incubated with intracellularly targeted antibodies for 30 min (anti-TCF1, -Ki67, -NKp46). MitoSOX Red staining was used to assess mitochondrial superoxide generation as an indicator of mitochondrial oxidative stress in live NK cells. Samples were acquired using a BD Fortessa flow cytometer and analyzed with DIVA software (BD Biosciences). Doublets were excluded from analysis using side-scatter (SSC) and forward-scatter (FSC) gating.

### Cell culture

Human NK cells (YT cells) and human HGSOC cells (CAOV3 cells and COV362 cells) were cultured in RPMI-1640 supplemented with 10% FBS, L-glutamine, and antibiotics. All cells were cultured at 37 °C in 5% CO₂ and periodically tested for mycoplasma. For co-culture experiments, CAOV3 or COV362 cells and allowed to adhere overnight at 37 °C in a 5% CO₂ incubator. The following day, non-adherent NK cells were harvested, washed, counted, and added to the adherent HGSOC monolayers at defined effector:target (E:T) ratios (5:1) in complete RPMI-1640 medium with 10% FBS. Phenotype of NK cells were checked at day 4 of co-culture using flow cytometry. All experiments were performed in triplicate and repeated independently at least three times.

### scRNAseq analysis

scRNA-seq data were obtained from the publicly available dataset GSE180661. Raw sequencing data were processed using the Seurat package (version 5.2.1) in R. The data were normalized using the SCTransform method, followed by principal component analysis (PCA) to reduce dimensionality. To visualize cellular heterogeneity, UMAP was applied using the top 30 principal components. Clustering was performed using the Louvain algorithm with a resolution parameter optimized based on silhouette scores and cluster stability assessment. A total of 15 distinct clusters were identified, corresponding to major immune cell populations, including T cells and NK cells. To specifically analyze NK cell populations, non-NK cell clusters were excluded, and re-clustering was performed on the remaining NK cell subsets. NK cell clusters were identified based on marker gene expression, including NKG7, KLRD1, NCAM1, and KLRF1. The expression of CD4 and CD8A was also examined to distinguish T cell populations from NK cells. Feature plots were generated to visualize marker gene expression across clusters, providing insights into the molecular heterogeneity of NK cells. Differential gene expression analysis was performed using the Wilcoxon rank-sum test to identify key regulatory genes distinguishing NK cell subpopulations.

### Cell deconvolution from RNA sequencing

Normalized transcriptomic data (FPKM) for HGSOC (TCGA-OV) were obtained from The Cancer Genome Atlas (TCGA). Gene identifiers in Ensembl format were truncated to remove version suffixes (e.g., ENSG00000123456.15 → ENSG00000123456) and mapped to official HGNC gene symbols using a custom dictionary derived from BioMart annotations. Genes without valid mappings were excluded from further analysis. To ensure consistency across datasets, sample identifiers were truncated to the first 15 characters. All FPKM values were log2-transformed using the formula log2(FPKM + 1) to stabilize variance and reduce skewness. To estimate the abundance of early immune cell states, transcriptional signatures for NK cells and CD8⁺ T cells were computed. For NK cells, the early NK score was defined as the average log-transformed expression of *TCF7* (TCF1), *NCAM1* (CD56), and *PTPRC (*CD45). For CD8⁺ T cells, the early/stem-like CD8 score was based on *TCF7*, *CD8A* (CD8), and PTPRC. All scores were calculated per sample by computing the arithmetic mean of the relevant genes’ expression values. Clinical metadata were downloaded from the TCGA data portal. Overall survival time was calculated using either days_to_death or days_to_last_follow_up, depending on patient vital status. Events were coded as 1 for deceased and 0 for alive. Patient barcodes were truncated to the first 12 characters to allow accurate merging with molecular data. Patients were stratified into “high” and “low” groups based on the median value of the early NK or early/stem-like CD8 score, respectively. Similarly, the early NK score was defined from bulkRNAseq results. Response status was encoded based on metadata embedded in the original expression file, with numeric values converted to categorical labels: '1' to 'responder' and '0' to 'nonresponder'. A merged annotation dataframe was generated linking each sample’s early NK score to its response classification. Kaplan–Meier survival curves were generated using the lifelines Python package, and statistical significance between survival curves was assessed with two-sided log-rank tests.

### Machine learning

To evaluate the predictive value of NK cell abundance in the context of specific mutational profiles in ovarian cancer, a machine learning approach was implemented separately for HGSOC and non-HGSOC subgroups. A curated dataset was used containing the proportion of NK % Intact Live cells per patient and binary mutation status for selected genes. For HGSOC, the mutational features included: *TP53*, *FRG1BP*, *COL22A1*, *NBPF14*, and *NBPF25P* (genes with *p* < 0.05 based on Fig. 2E). For non-HGSOC, the selected mutations were: *TTN*, *NBPF1*, *NBPF8*, *RGPD3*, *NBPF10*, *NBPF12*, and *SRSF3P2* (genes with p < 0.05 based on Fig. 2F). Each group also included the NK % Intact Live feature as a baseline immune-related measure. For every mutation feature, interaction terms were generated by multiplying the NK % Intact Live value with the respective mutation indicator, resulting in interaction variables such as NKxTP53, NKxFRG1BP, etc. Random Forest classifiers were trained for each group using three strategies: (1) individual features, (2) all pairwise feature combinations, and (3) all features simultaneously. Model performance was evaluated via stratified cross-validation (fivefold for HGSOC, threefold for non-HGSOC), with the AUC as the scoring metric. For each model, mean AUC and standard deviation were recorded across folds. To further explore associations between mutational status and immune abundance, pairwise statistical comparisons were performed using the Mann–Whitney U test. All data processing, machine learning, and visualization steps were conducted in Python using the pandas, numpy, scikit-learn, scipy, seaborn, and matplotlib libraries.

### Bioinformatics analyses

Publicly available previously generated scRNAseq and RNAseq were obtained from the Gene Expression Omnibus database with the accession numbers (GSE180661 and GSE63885, respectively). Data of The Cancer Genome Atlas ovarian cancer data collection (TCGA-OV) were obtained from the Genomic Data Commons (GDC) (National Cancer Institute).

### Statistical analyses

Statistical analyses were carried out with a t-test. Error bars in data represent SEM. Experiments were analyzed using Prism6 (GraphPad Software) and Python. Graphics were prepared using BioRender. CyTOF data were analyzed using Maxpar Pathsetter 3.0 and Cytobank. For scRNAseq data analysis and machine learning packages in R (version 4.4.1) and Python were used.

## Supplementary Information

Supplementary Material 1.

Supplementary Material 2.

## Data Availability

All data supporting the results of this study are available from the corresponding author upon reasonable request.

## References

[1] Kandalaft LE, Dangaj Laniti D, Coukos G. Immunobiology of high-grade serous ovarian cancer: lessons for clinical translation. Nat Rev Cancer. 2022;22:640–56. 10.1038/s41568-022-00503-z. 36109621 10.1038/s41568-022-00503-z

[2] Ghisoni E, Morotti M, Sarivalasis A, Grimm AJ, Kandalaft L, Laniti DD, et al. Immunotherapy for ovarian cancer: towards a tailored immunophenotype-based approach. Nat Rev Clin Oncol. 2024;21:801–17. 10.1038/s41571-024-00937-4. 39232212 10.1038/s41571-024-00937-4

[3] Rajtak A, Ostrowska-Leśko M, Żak K, Tarkowski R, Kotarski J, Okła K. Integration of local and systemic immunity in ovarian cancer: implications for immunotherapy. Front Immunol. 2022. 10.3389/fimmu.2022.1018256 36439144 10.3389/fimmu.2022.1018256 PMC9684707

[4] Ray-Coquard I, Leary A, Pignata S, Cropet C, González-Martín A, Marth C, et al. Olaparib plus Bevacizumab first-line maintenance in ovarian cancer: final overall survival results from the PAOLA-1/ENGOT-Ov25 trial. Ann Oncol. 2023;34:681–92. 10.1016/j.annonc.2023.05.005. 37211045 10.1016/j.annonc.2023.05.005

[5] Moore K, Colombo N, Scambia G, Kim B-G, Oaknin A, Friedlander M, et al. Maintenance olaparib in patients with newly diagnosed advanced ovarian cancer. N Engl J Med. 2018;379:2495–505. 10.1056/NEJMoa1810858. 30345884 10.1056/NEJMoa1810858

[6] Ribas A, Wolchok JD. Cancer immunotherapy using checkpoint blockade. Science. 2018;359:1350–5. 10.1126/science.aar4060. 29567705 10.1126/science.aar4060 PMC7391259

[7] Lheureux S, Braunstein M, Oza AM. Epithelial ovarian cancer: evolution of management in the era of precision medicine. CA Cancer J Clin. 2019;69:280–304. 10.3322/caac.21559. 31099893 10.3322/caac.21559

[8] Okła K. Myeloid-derived suppressor cells (MDSCs) in ovarian cancer—looking back and forward. Cells. 2023;12:1912. 10.3390/cells12141912. 37508575 10.3390/cells12141912 PMC10377883

[9] Rajtak A, Czerwonka A, Pitter M, Kotarski J, Okła K. Clinical relevance of mortalin in ovarian cancer patients. Cells. 2023;12:701. 10.3390/cells12050701. 36899836 10.3390/cells12050701 PMC10000941

[10] Okła K, Farber DL, Zou W. Tissue-resident memory T cells in tumor immunity and immunotherapy. J Exp Med. 2021;218:e20201605. 10.1084/jem.20201605. 33755718 10.1084/jem.20201605 PMC7992502

[11] Bian Y, Li W, Kremer DM, Sajjakulnukit P, Li S, Crespo J, et al. Cancer SLC43A2 alters T cell methionine metabolism and histone methylation. Nature. 2020;585:277–82. 10.1038/s41586-020-2682-1. 32879489 10.1038/s41586-020-2682-1 PMC7486248

[12] Pitter MR, Kryczek I, Zhang H, Nagarsheth N, Xia H, Wu Z, et al. PAD4 controls tumor immunity via restraining the MHC class II machinery in macrophages. Cell Rep. 2024;43:113942. 10.1016/j.celrep.2024.113942. 38489266 10.1016/j.celrep.2024.113942 PMC11022165

[13] Cui TX, Kryczek I, Zhao L, Zhao E, Kuick R, Roh MH, et al. Myeloid derived suppressor cells enhance stemness of cancer cells by inducing microrna101 and suppressing the corepressor CtBP2. Immunity. 2013. 10.1016/j.immuni.2013.08.025. 24012420 10.1016/j.immuni.2013.08.025 PMC3831370

[14] Wang W, Kryczek I, Dostál L, Lin H, Tan L, Zhao L, et al. Effector t cells abrogate stroma-mediated chemoresistance in ovarian cancer. Cell. 2016;165:1092–105. 10.1016/j.cell.2016.04.009. 27133165 10.1016/j.cell.2016.04.009 PMC4874853

[15] Peng D, Kryczek I, Nagarsheth N, Zhao L, Wei S, Wang W, et al. Epigenetic silencing of TH1-type chemokines shapes tumour immunity and immunotherapy. Nature. 2015;527:249–53. 10.1038/nature15520. 26503055 10.1038/nature15520 PMC4779053

[16] Zhao E, Maj T, Kryczek I, Li W, Wu K, Zhao L, et al. Cancer mediates effector T cell dysfunction by targeting micrornas and EZH2 via glycolysis restriction. Nat Immunol. 2016;17:95–103. 10.1038/ni.3313. 26523864 10.1038/ni.3313 PMC4684796

[17] Burdett NL, Willis MO, Alsop K, Hunt AL, Pandey A, Hamilton PT, et al. Multiomic analysis of homologous recombination-deficient end-stage high-grade serous ovarian cancer. Nat Genet. 2023;55:437–50. 10.1038/s41588-023-01320-2. 36849657 10.1038/s41588-023-01320-2

[18] Vázquez-García I, Uhlitz F, Ceglia N, Lim JLP, Wu M, Mohibullah N, et al. Ovarian cancer mutational processes drive site-specific immune evasion. Nature. 2022;612:778–86. 10.1038/s41586-022-05496-1. 36517593 10.1038/s41586-022-05496-1 PMC9771812

[19] Mapping Spatial Organization and Genetic Cell-State Regulators to Target Immune Evasion in Ovarian Cancer - PMC Available online: https://pmc.ncbi.nlm.nih.gov/articles/PMC11436371/ (Accessed on 19 Aug 2025). 10.1038/s41590-024-01943-5 PMC11436371 39179931

[20] Zhu K, Su F, Yang J, Xiao R, Wu R, Cao M, et al. TP53 to mediate immune escape in tumor microenvironment: an overview of the research progress. Mol Biol Rep. 2024;51:205. 10.1007/s11033-023-09097-7. 38270700 10.1007/s11033-023-09097-7 PMC10811008

[21] Wang C, Tan JYM, Chitkara N, Bhatt S. TP53 mutation-mediated immune evasion in cancer: mechanisms and therapeutic implications. Cancers (Basel). 2024;16:3069. 10.3390/cancers16173069. 39272927 10.3390/cancers16173069 PMC11393945

[22] Wang H, Chen Q, Liu Q, Luo C. Master regulator: P53’s pivotal role in steering NK-cell tumor patrol. Front Immunol. 2024;15:1428653. 10.3389/fimmu.2024.1428653. 39185404 10.3389/fimmu.2024.1428653 PMC11344261

[23] Gupta R, Kumar R, Penn CA, Wajapeyee N. Immune evasion in ovarian cancer: implications for immunotherapy and emerging treatments. Trends Immunol. 2025;46:166–81. 10.1016/j.it.2024.12.006. 39855990 10.1016/j.it.2024.12.006 PMC11835538

[24] Okła K, Czerwonka A, Wawruszak A, Bobiński M, Bilska M, Tarkowski R, et al. Clinical relevance and immunosuppressive pattern of circulating and infiltrating subsets of Myeloid-Derived Suppressor Cells (MDSCs) in epithelial ovarian cancer. Front Immunol. 2019. 10.3389/fimmu.2019.00691. 31001284 10.3389/fimmu.2019.00691 PMC6456713

[25] Ponton-Almodovar A, Sanderson S, Rattan R, Bernard JJ, Horibata S. Ovarian tumor microenvironment contributes to tumor progression and chemoresistance. Cancer Drug Resist. 2024;7:53. 10.20517/cdr.2024.111. 39802952 10.20517/cdr.2024.111 PMC11724355

[26] Xu X, Li R, Mo O, Liu K, Li J, Hao P. Cell-type deconvolution for bulk RNA-seq data using single-cell reference: a comparative analysis and recommendation guideline. Brief Bioinform. 2025;26:bbaf031. 10.1093/bib/bbaf031. 10.1093/bib/bbaf031 PMC11789683 39899596

[27] Liu J, Wang Z, Hao S, Wang F, Yao Y, Zhang Y, et al. Tcf1 sustains the expression of multiple regulators in promoting early natural killer cell development. Front Immunol. 2021;12:791220. 10.3389/fimmu.2021.791220. 34917097 10.3389/fimmu.2021.791220 PMC8669559

[28] Myers JA, Schirm D, Bendzick L, Hopps R, Selleck C, Hinderlie P, et al. Balanced engagement of activating and inhibitory receptors mitigates human NK cell exhaustion. JCI Insight. 2022;7:e150079. 10.1172/jci.insight.150079. 35727627 10.1172/jci.insight.150079 PMC9462468

[29] Jeevan-Raj B, Gehrig J, Charmoy M, Chennupati V, Grandclément C, Angelino P, et al. The transcription factor Tcf1 contributes to normal NK cell development and function by limiting the expression of granzymes. Cell Rep. 2017;20:613–26. 10.1016/j.celrep.2017.06.071. 28723565 10.1016/j.celrep.2017.06.071

[30] Binnewies M, Roberts EW, Kersten K, Chan V, Fearon DF, Merad M, et al. Understanding the tumor immune microenvironment (TIME) for effective therapy. Nat Med. 2018;24:541–50. 10.1038/s41591-018-0014-x. 29686425 10.1038/s41591-018-0014-x PMC5998822

[31] Ding K, Zhou M, Wang H, Gevaert O, Metaxas D, Zhang S. A large-scale synthetic pathological dataset for deep learning-enabled segmentation of breast cancer. Sci Data. 2023;10:231. 10.1038/s41597-023-02125-y. 37085533 10.1038/s41597-023-02125-y PMC10121551

[32] Forks in the Road for CAR T and CAR NK Cell Cancer Therapies | Nature Immunology Available online: https://www.nature.com/articles/s41590-023-01659-y. (Accessed on 19 Aug 2025). 10.1038/s41590-023-01659-y PMC12798859 38012406

[33] Wang W, Liu Y, He Z, Li L, Liu S, Jiang M, et al. Breakthrough of solid tumor treatment: CAR-NK immunotherapy. Cell Death Discov. 2024;10:40. 10.1038/s41420-024-01815-9. 10.1038/s41420-024-01815-9 PMC10799930 38245520

[34] CAR-T and CAR-NK as Cellular Cancer Immunotherapy for Solid Tumors | Cellular & Molecular Immunology Available online: https://www.nature.com/articles/s41423-024-01207-0. (Accessed on 19 Aug 2025). 10.1038/s41423-024-01207-0 PMC11442786 39134804

