# Supplementary material for: Integrative and deep learning-based prediction of therapy response in ovarian cancer
Source: J Exp Clin Cancer Res. 2025 Nov 28;44:313. doi: 10.1186/s13046-025-03554-w (PMC12661774; doi:10.1186/s13046-025-03554-w)
Supplement: Supplementary file 2 — Supplementary Material 2. [file 13046_2025_3554_MOESM2_ESM.pdf]

Fig. S1. Overview of the study design

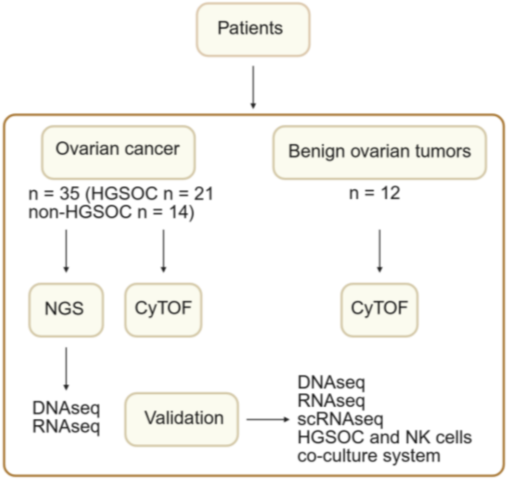

Fig. S2. Genomic alterations predict treatment outcomes beyond clinical features

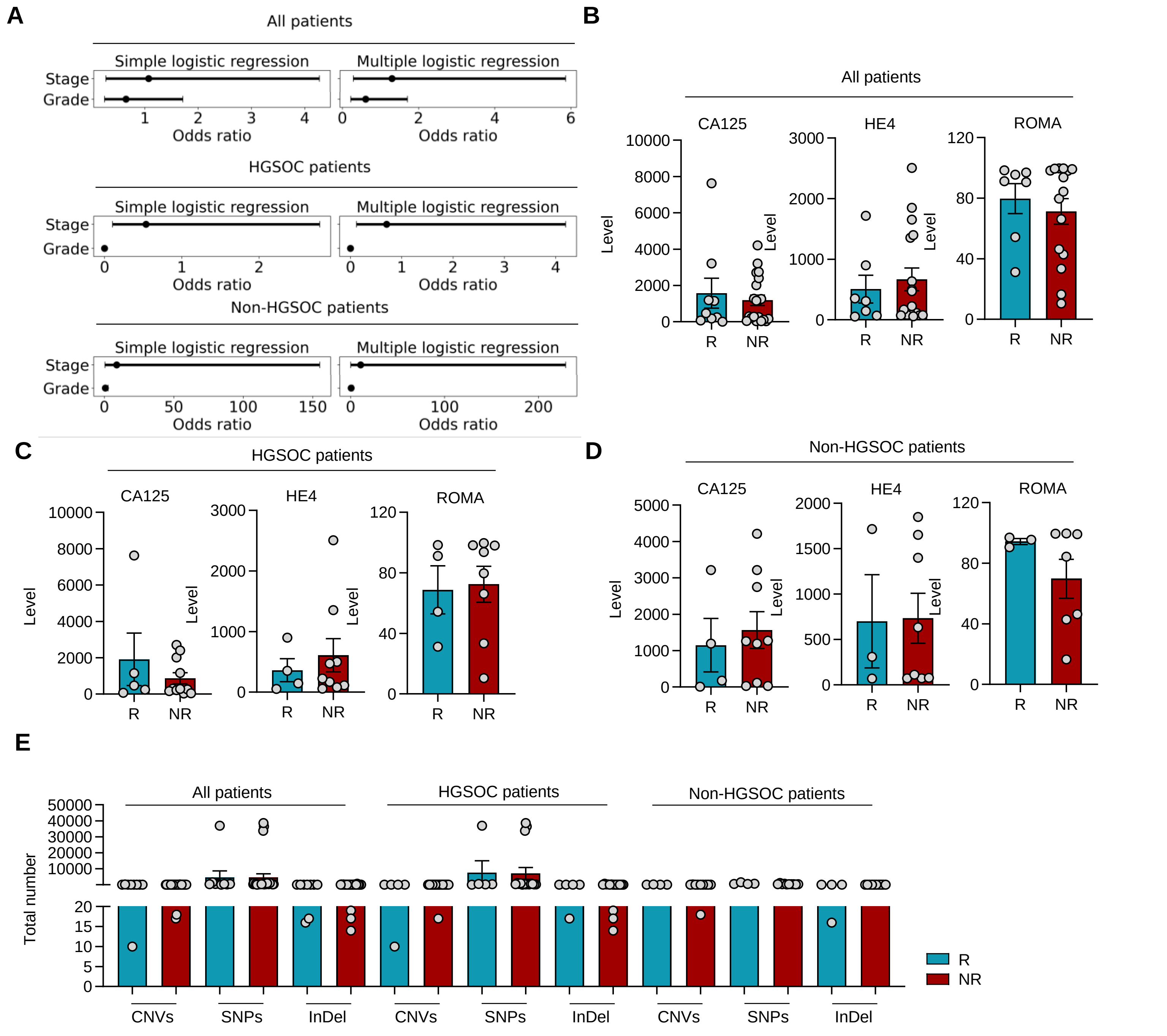

Fig. S3. TME landscapes in ovarian tumors

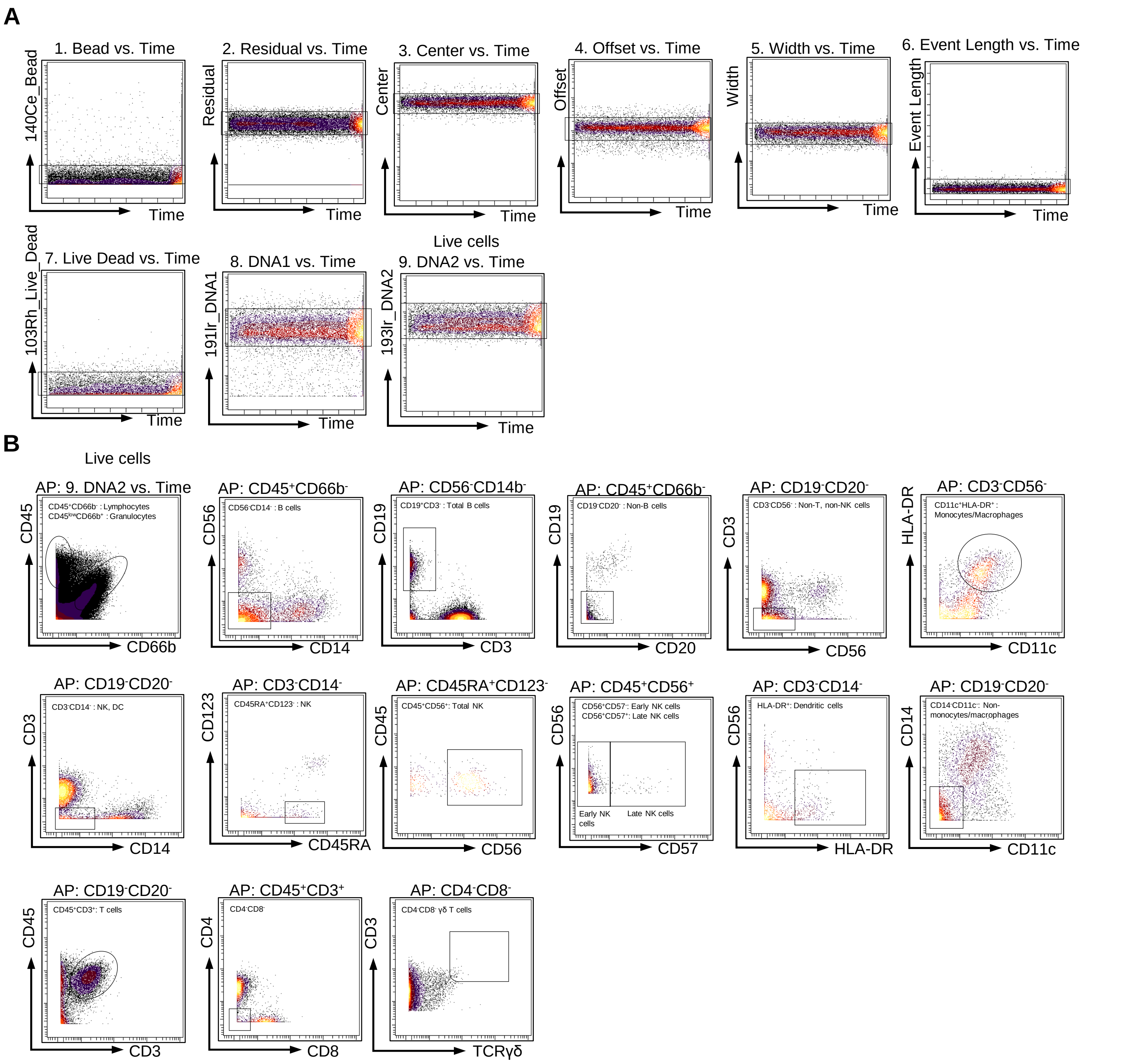

Fig. S4. Tumor early NK cells dictate clinical outcome in ovarian cancer

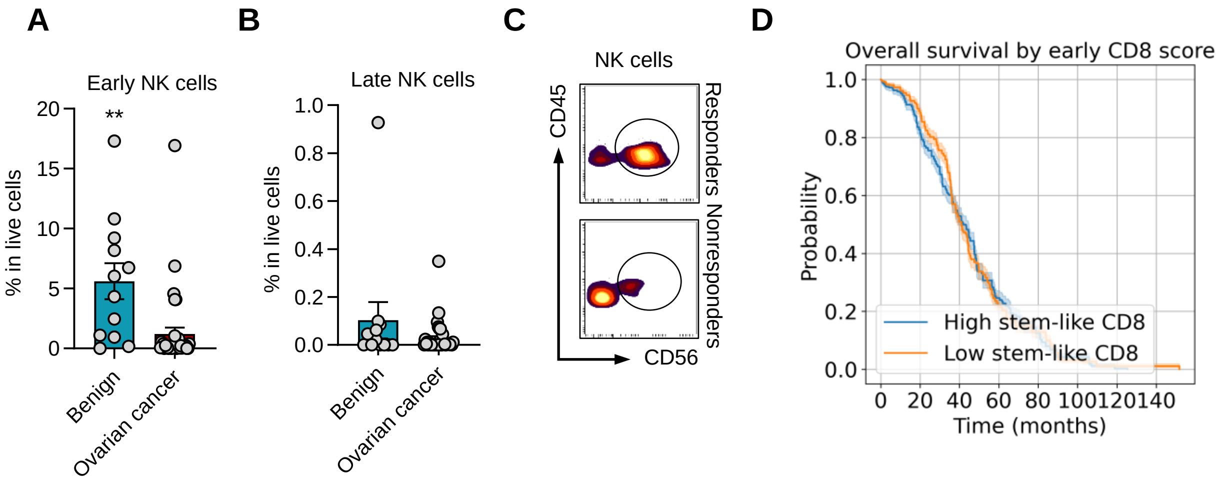

Fig. S5. Tumor early NK cells show persistent phenotype

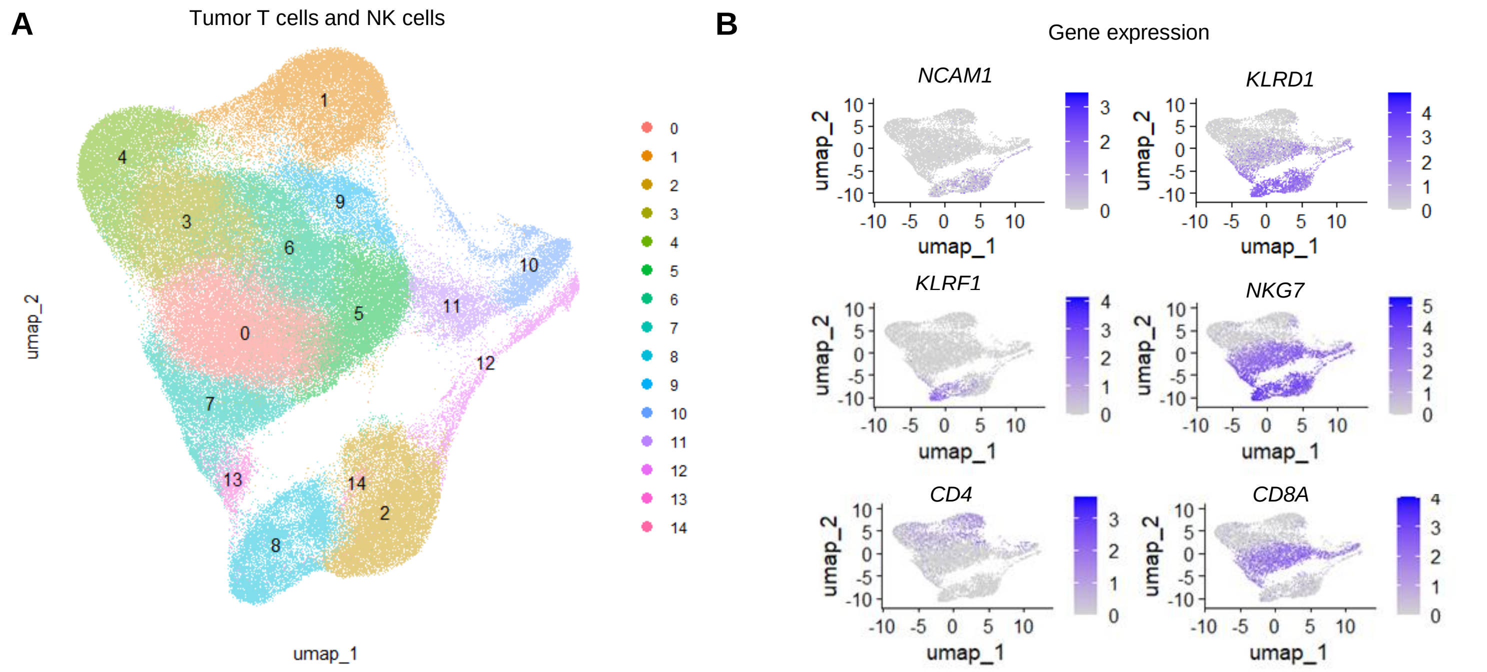

Fig. S6. Integrating composite machine learning techniques to enhance therapy response predictions.

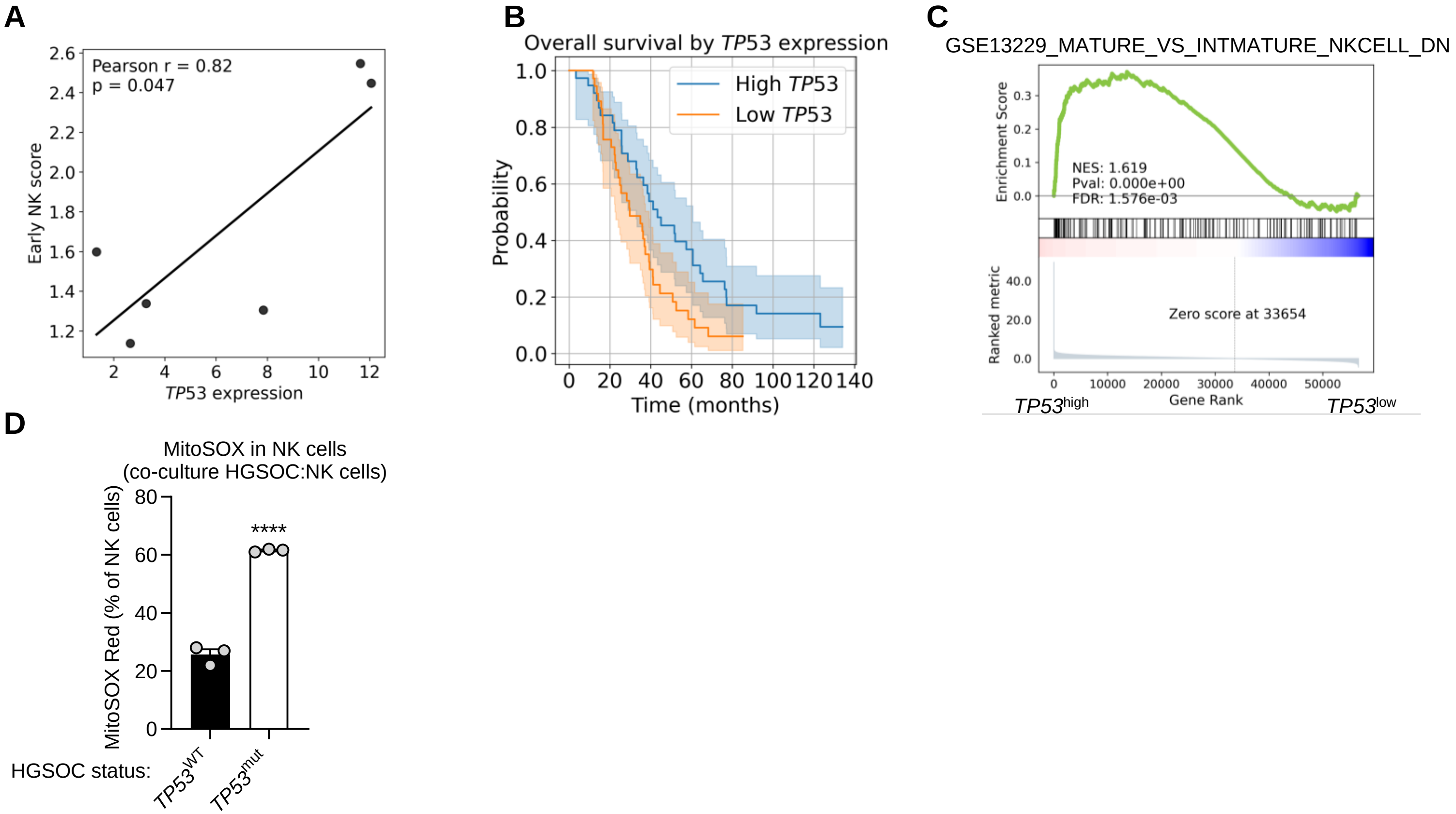

Figure S1. Overview of the study design.

The diagram illustrates the classification of patients in the study into two groups: those with ovarian cancer (n = 35; including high grade serous ovarian cancer (HGSOC) n = 21 and non-HGSOC n = 14) and those with benign ovarian tumors (n = 12). In the ovarian cancer group, analysis was performed using next-generation sequencing (NGS) and mass cytometry (CyTOF). In the benign ovarian tumor group, analysis was conducted using CyTOF. For validation, DNA sequencing (DNAseq), RNA sequencing (RNAseq), single-cell RNA sequencing (scRNAseq) and high grade serous ovarian cancer (HGSOC) and natural killer (NK) cells co-culture system were used.

Figure S2. Genomic alterations predict treatment outcomes beyond clinical features.

(A) Simple and multiple logistic regression analyses were performed to evaluate the associations of clinical variables such as stage and grade with therapy response (R; responders, NR; nonresponders) across all patients, high grade serous ovarian cancer (HGSOC) patients and non-HGSOC patients. The odds ratios were calculated and plotted to indicate the significance of these factors (n = 35).

(B-D) The levels of clinical biomarkers CA125, HE4 and ROMA across all patients (B), high grade serous ovarian cancer (HGSOC) patients (C) and non-HGSOC patients (D) were measured and compared between responders and nonresponders. The y-axis represents the biomarker levels, while the x-axis distinguishes between the two groups (n = 35).

(E) The numbers of somatic copy number variations (CNVs), single nucleotide polymorphisms (SNPs) and insertions and deletions (InDel) in various genomic regions were analyzed and compared between responders and nonresponders across all patients, high grade serous ovarian cancer (HGSOC) patients and non-HGSOC patients (n = 35).

Figure S3. TME landscapes in ovarian tumors.
(A) Cleaning strategy for mass cytometry (CyTOF). Each cleanup parameter is plotted against time. The gates are adjusted to remove aggregates, debris, beads, doublets, and dead cells. Cleanup_DNA2 population is the live singlet population.
(B) Gating strategy for CyTOF. For cell analysis Maxpar Pathsetter 3.0 was used.

Figure S4. Tumor early NK cells dictate clinical outcome in ovarian cancer.
(A and B) Mass cytometry (CyTOF) analysis of early NK cells (A) and late NK cells (B) was compared between benign ovarian tumors and ovarian cancer ($n = 47$, mean ± SEM, $^{**}p < 0.01$, unpaired t-test).
(C) CyTOF analysis and representative density plot of NK cells in responders and nonresponders.
(D) Kaplan-Meier survival curve evaluates the impact of high and low stem-like CD8 cell score in HGSOC patients using data from the TCGA-OV dataset ($n = 429$, log-rank $p = 0.00003$). Stem-like CD8 cell score was assessed using deconvolution method.

Figure S5. Tumor early NK cells show persistent phenotype.

(A) Uniform manifold approximation and projection (UMAP) plot is presented, showing the clustering of T cells and NK cells into 15 distinct groups, each represented by a different color. This dimensionality reduction approach was used to visualize cellular heterogeneity and relationships among different immune cell populations (n = 42 HGSOC patients, GSE180661).

(B) Six smaller UMAP plots display the expression levels of key surface markers across the identified cell populations: *NCAM1*, *KLRD1*, *KLRF1*, *NKG7* (markers for NK cells), *CD4* and *CD8A* (marker for T cells).

Figure S6. Integrating composite machine learning techniques to enhance therapy response predictions.
(A) Correlation of *TP53* expression with early NK cell score across HGSOC patients (n = 6). HGSOC tissues were sequenced (RNAseq) and early NK score was assessed using deconvolution method.
(B) Kaplan-Meier survival curve evaluates the impact of high and low *TP53* expression in ovarian cancer patients (GSE63885, n = 75, log-rank p = 0.019).
(C) Gene set enrichment analysis (GSEA) plot showing enrichment of the MATURE_VS_INTMATURE_NKCELL signature between *TP53*-high and *TP53*-low expression groups (n = 429, TCGA-OV).
(D) Flow cytometry analysis of MitoSOX in natural killer (NK) cells. Human NK cells (YT) were co-cultured with high grade serous ovarian cancer (HGSOC) cells (TP53WT; wild type TP53 (CAOV3) and TP53mut; mutated TP53 (COV362)) (n = 6, mean ± SEM, ****p < 0.00001, unpaired t-test, one from three experiments is shown).
